# Exploring blood microbial communities and their influence on human cardiovascular disease

**DOI:** 10.1002/jcla.24354

**Published:** 2022-03-15

**Authors:** Ikram Khan, Imran Khan, Zhou Jianye, Zhang Xiaohua, Murad Khan, Mian Gul Hilal, Mian Adnan Kakakhel, Arshad Mehmood, An Lizhe, Li Zhiqiang

**Affiliations:** ^1^ 12426 School of Life Sciences Lanzhou University Lanzhou China; ^2^ Department of Microbiology Khyber Medical University Peshawar Peshawar Pakistan; ^3^ 66293 Key Laboratory of Oral Diseases of Gansu Province School of Stomatology Northwest Minzu University Lanzhou China; ^4^ 12553 Department of Genetics Hebei Key Laboratory Animal Hebei Medical University Shijiazhuang China; ^5^ 71213 Department of Neurology The Second Hospital of Hebei Medical University Shijiazhuang China

**Keywords:** bacteria, bacterial translocation, blood circulation, cardiovascular diseases, noncommunicable diseases

## Abstract

**Background:**

Cardiovascular disease (CVD) is the single biggest contributor to global mortality. CVD encompasses multiple disorders, including atherosclerosis, hypertension, platelet hyperactivity, stroke, hyperlipidemia, and heart failure. In addition to traditional risk factors, the circulating microbiome or the blood microbiome has been analyzed recently in chronic inflammatory diseases, including CVD in humans.

**Methods:**

For this review, all relevant original research studies were assessed by searching in electronic databases, including PubMed, Google Scholar, and Web of Science, by using relevant keywords.

**Results:**

This review demonstrated that elevated markers of systemic bacterial exposure are associated with noncommunicable diseases, including CVD. Studies have shown that the bacterial DNA sequence found in healthy blood belongs mainly to the Firmicutes, Bacteroidetes, Proteobacteria, and Actinobacteria phyla. In cardiac events, such as stroke, coronary heart disease, and myocardial infarction, the increased proportion of Proteobacteria and Actinobacteria phyla was found. Lipopolysaccharides are a major component of Proteobacteria, which play a key role in the onset of CVD. Moreover, recently, a study reported the lower cholesterol‐degrading bacteria, including Caulobacterales order and Caulobacteraceae family were both considerably reduced in myocardial infarction.

**Conclusion:**

Proteobacteria and Actinobacteria were shown to be independent markers of the risk of CVD. This finding is evidence for the new concept of the role played by blood microbiota dysbiosis in CVD. However, the association between blood microbiota and CVD is still inconsistent. Thus, more deep investigations are required in future to fully understand the role of the bacteria community in causing and preventing CVD.

## Graphical abstract

In this study, we highlight the origin of the blood microbiota, alteration in the blood microbial makeup, and their association with the etiology and progression of cardiovascular disease.

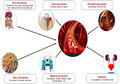

## 1 INTRODUCTION

Despite the widespread use of medical therapy, cardiovascular disease (CVD) is still the leading cause of morbidity and mortality globally.^1^ CVD comprises cardiomyopathies, heart failure (HF), congenital heart disease (CHD), coronary artery disease (CAD), cerebrovascular disease, abnormal heart rhythms, peripheral artery disease (PAD), aortic disease, valvular heart disease (VHD), rheumatic heart disease (RHD),^2^ and several other cardiac and vascular conditions.^3^ Each year, millions of people die due to CVD, which coincides with approximately 31% of deaths occurring globally and accounting for the cost of approximately one trillion dollars per year.^4^ In 2017, CVD, including stroke and ischemic heart diseases, accounted for 17.8 million deaths worldwide, representing approximately 32% of total global deaths, with three‐quarters of these deaths in low‐ and middle‐income countries.^3^, ^5^ In addition, one out of every three deaths in the United States (US) and one out of every four deaths in Europe and Japan are caused by CVDs.^6^ Between 2010 and 2030, 40.5% of the US population is expected to have some sort of CVD, with real overall direct medical costs of CVDs expected to triple from 273 billion dollars to 818 billion dollars, and real indirect costs (due to lost productivity) for all CVDs expected to rise by 61% (172 billion to 276 billion dollars).^7^ Moreover, a recent study demonstrated that the highest CVD deaths were reported in China during the last 5 years, followed by India, Russia, the US, and Indonesia, as depicted.^5^ Several risk factors play a role in the progression of CVD, such as genetics, epigenetics, and lifestyles; the microbial population is known as microbiota and is another factor that has lately been studied.^8^

The human microbiota is a collection of trillions of different microbes that inhabit the human body^9^; these microbiotas live in symbiosis with a wide range of communities, which are thought to have an impact on health and disease.^10^ Previously, the blood was thought to be immune‐privileged, and microbial infiltration into the blood was only associated with infectious diseases.^11^ Over the last decade, this perception has been shattered by studies reporting circulating microbial metabolites and the presence of the blood microbiota.^12^ Microorganisms in blood have been discovered to impact host immune regulation and chronic inflammatory diseases such as CVDs.^13^ The existence of circulating microbiota was initially discovered in a large group study consisting of diabetes mellitus and obese individuals.^14^ Surprisingly, the patients who had diabetes mellitus and individuals who had a risk of diabetes mellitus both had a similar blood microbiota. The blood microbiome of individuals with end‐stage renal disease (ESRD) was then profiled.^15^ Intestinal microbiota was found in the blood of ESRD subjects, who also had higher levels of inflammatory markers in their plasma, such as interleukin‐6, lactate, and high sensitivity C‐reactive protein, indicating that intestinal microbiota migration is linked to proinflammation in ESRD patients.

Moreover, for the first time, the existence of the circulating microbiome and its long‐term prognosis in patients with CVD was investigated, with the finding that disruptions in blood microbiota equilibrium are related to or associated with the onset of the disease.^16^ Following that, a study on the existence of blood microbiota in CVD patients came up with similar results^17^; because these studies were preliminary, the findings must be confirmed in larger research with a large samples size to demonstrate the existence of a stable circulating microbiota in the following infections. Bacterial DNA might be an easy victim in the disease progression. The tools governing circulating microbiota synergy and the molecular interplay among bacteria and host needed to be further investigated.

Based on the previous studies, Proteobacteria were shown to be the most common blood microbial community in CVD. These results suggest that the existence of a circulating microbiota might be one of the primary steps in the development of metabolic diseases like diabetes, CVD, and obesity. The existence of stable circulating microbes in various disorders might be demonstrated using this preliminary data. The role of inflammation in the progression of many diseases, and the proinflammatory consequences of bacterial blood components, may suggest that these bacteria are involved in the disease‐clinical outcomes. The influence of the intestinal microbiome on innate immunity and CVD complication has been recently investigated.^18^ In addition, the role of oral microbiota in the etiology of various disorders has been extensively studied.^19^ Circulating microbiome studies give the first evidence of tissue microbial involvement in various disorders, and some insight into the mechanism of action of the blood microbiome. These investigations were observational, and they were unable to show that blood microbiota played a causal or important role in the genesis of these disorders. However, the specifics and relevance of the blood microbial profile in human health are just beginning to be explored. Thus, this review highlights the evidence for a relationship between the blood microbiota and CVDs to facilitate a better understanding of the most current perspectives on the blood microbiota composition and CVD.

## 2 HISTORY OF BLOOD MICROBIOTA

**FIGURE 1:**
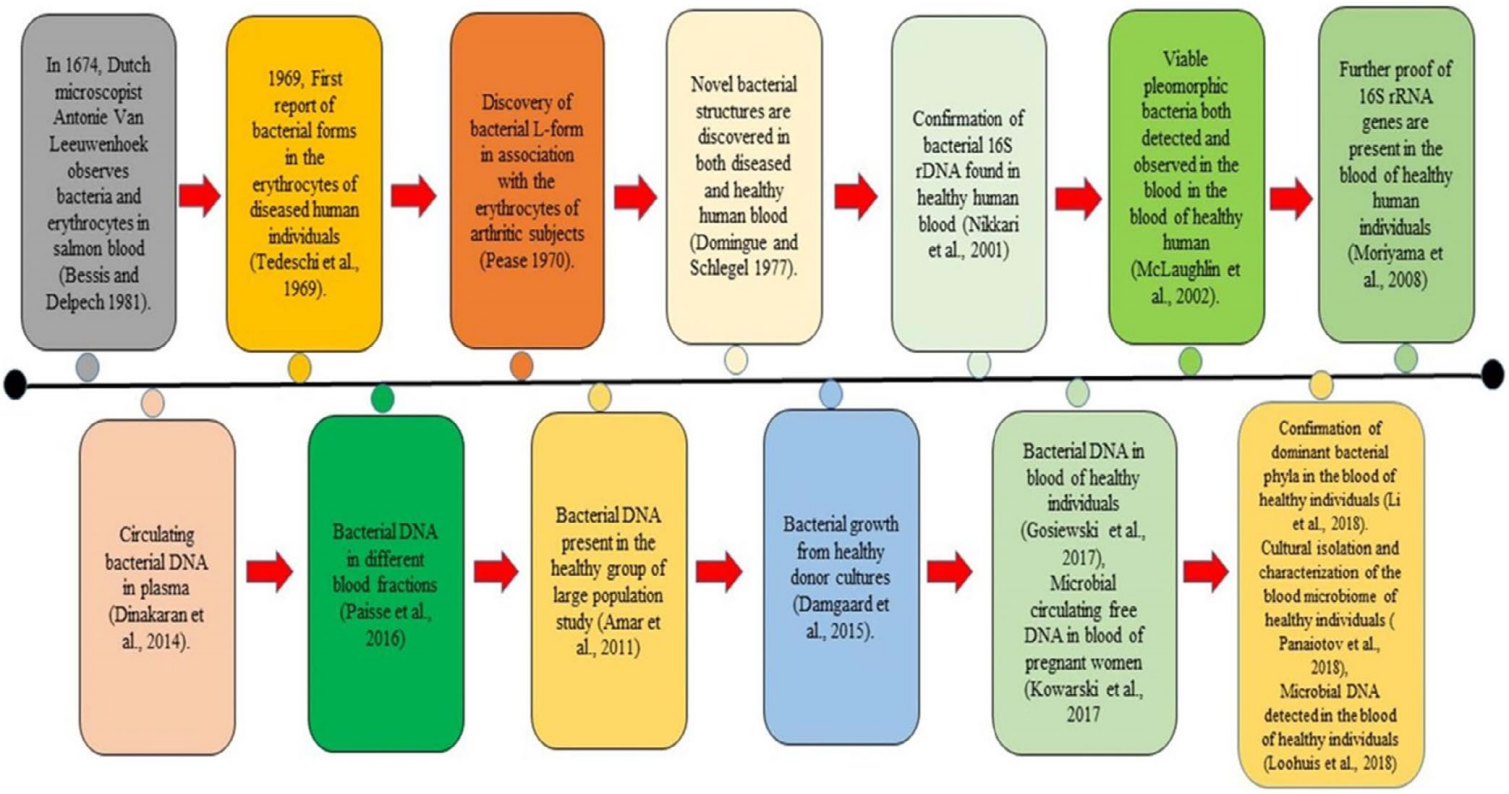
Timeline indicating significant advances concerning human blood microbiome research

Blood has long been thought to be a germ‐free zone, but later portable microparticles detected in the blood were named “animalcules.” In 1674, Anton Van Leuwenhoek presented the primary microscopic study of red blood cells (RBC) and minute organisms in salmon blood. Robert Koch observed in 1876 that blood microorganisms might cause disease, as shown in Figure 1. During his research, he discovered a large number of Bacillus anthracis in the blood of anthrax‐infected cattle. After that, Koch took blood from anthrax‐infected cattle and inserted it into healthy cattle by making the healthy cattle infected, revealing how anthrax spread from one to another animal. Blood microbes were believed to be pathogenic and only related to infectious diseases. In the mid‐twentieth century, bacteria were discovered in the blood using culture techniques, even in healthy individuals' blood. First, the detection of bacterial DNA from healthy human blood was reported.^20^ As a result, researchers have found living microorganisms or bacterial DNA in both healthy and diseased individuals, and their association with diseases such as asthma, Alzheimer's disease (AD), liver cirrhosis, liver fibrosis, Parkinson's disease (PD), rosacea skin infection, diabetes, and CVD.^21^, ^22^

The presence of archaeal DNA and viruses in the circulation was also shown by Shotgun sequencing of the blood metagenome. Patients had a higher prevalence of bacteriophages, whereas healthy people had a higher prevalence of viruses.^17^ In the blood of healthy humans, a study using ITS2 targeted sequencing discovered the existence of a fungal microbiome influenced by two fungus taxa (Basidiomycota and Ascomycota). Advanced metabolic technologies, particularly the 2011 study on trimethylamine N‐oxide (TMAO), made it possible to identify microbial metabolites in circulation.^23^ It was a watershed moment in the research of microbial circulating metabolites and disease.

## 3 HEALTHY HUMAN BLOOD MICROBIOTA

The 16S rRNA detection by sequencing has been regarded as a hallmark of microbial existence and a means to quantify bacterial diversity since the emergence of next‐generation sequencing (NGS)‐based technology. However, in recent years, several researchers have concentrated their efforts on detecting 16S rRNA in a sterile zone, such as the blood of healthy individuals.^24^ Previous studies found bacterial 16S rRNA in healthy human blood, but the ultimate mechanisms and existence of the bacteria were not investigated.^20^ Bacterial diffusion from the mother before birth or migration from other organs during the ordinary lifespan has been the only explanation.^24^ Several studies showed that the phylum Proteobacteria dominates the blood microbiota of healthy humans, followed by Actinobacteria, Bacteroidetes, and Firmicutes, though there were variations between studies.^11^ Paisse et al. investigated the blood of thirty young, healthy volunteers in different fractions such as DNA extracted from RBC, buffy coat, and whole blood by sequencing 16S rRNA gene V3‐V4 hypervariable regions to investigate the blood microbiome composition using qPCR. The buffy coat had the highest percentage of bacterial DNA (93.74%), followed by RBCs (6.23%) and plasma (0.03%), RBC fraction containing greater bacterial diversity than a buffy coat and plasma.^25^ Intriguingly, Proteobacteria were found in more than 80% of the blood samples, followed by Actinobacteria (6.7%–10%) depending on the fraction, in contrast to the gut taxa Firmicutes and Bacteroidetes.^26^ These studies revealed that the blood microbiota could be translocated from the gastrointestinal tract (GIT) because most of the bacteria, as mentioned earlier at the phylum level, have been reported in the gut in several previous studies.^27^, ^28^

According to recent studies, microbial DNA is mainly found in cellular components in healthy people^25^; there was a significant difference between the healthy gut and the blood microbial community composition. Firmicutes and Bacteroidetes phyla were predominant in the gut, while Proteobacteria was found dominant in the blood. The blood microbiota is more intimately associated with the skin and oral microbiota. Studies on healthy blood suggest that more bacterial translocation occurs from these niches under normal physiology rather than the gut. Several studies have discovered viruses, fungi, and archaea in the blood of healthy people.^11^ The healthy human circulating microbes are assumed to be inactive since it does not cause intricacies like sepsis and inflammation; however, the circulating microbiome performs a vital function in natural physiology and immunology.^29^ Moreover, further study is necessary to explore the role of blood microbiota, its physiology, and immunity in healthy humans.

## 4 BLOOD MICROBIOTA AND DISEASE

As distinct anatomical areas of the human body have their microbiota, dysbiosis in its composition is bound to cause and/or contribute to a variety of human diseases.^30^ Many studies have found that dysbiosis has been linked to the onset and development of a variety of diseases.^31^ For instance, the correlation between the circulating microbiome and the development of liver disease has been revealed by different studies.^32^ In addition, the researchers used 16S rRNA sequencing and qPCR to evaluate the link between circulating microbial community profiles and liver fibrosis in obese persons.^33^ Patients with fibrosis revealed increased levels of 16S rRNA in their blood than healthy people, indicating that there is a unique microbial collection linked to liver fibrosis that might be used as a probe for initial diagnosis.^34^ In addition, a previous study evaluates the relation between alteration in the circulating microbiome and Parkinson's disease. The 16S rRNA sequencing was used to evaluate the taxonomic composition, where some taxa were found that related to pathology, including Cloacibacterium, Isoptericola, Paludibacter, and Saccharofermentans.^35^ Hammad and colleagues have evaluated the characteristics of blood bacterial DNA in ankylosing spondylitis (AS), rheumatoid arthritis (RA) patients, psoriatic arthritis (PA), and controls. In this regard, to identify bacterial community members, the 16S rRNA V4 region was sequenced in all samples, the blood bacteria was dominated by four phyla, mainly Proteobacteria, Actinobacteria, Firmicutes, and Bacteroidetes, and at the phylum level, confirming the prior findings of a core blood microbiome.^36^

Moreover, another study examined the microbial signature without ascites, and ascite patients and various and unique microbial communities were identified in the ascitic fluid and serum of cirrhotic patients using high‐throughput 16S rDNA sequencing. Surprisingly, microorganisms were virtually completely absent in sera taken from healthy controls.^37^ The researchers discovered an unknown phylum of Cyanobacteria in the serum of people suffering from ascites.

Additionally, the blood‐associated microbiome has been studied in dermatologic diseases such as hidradenitis suppurativa,^38^ psoriasis,^39^ and rosacea.^40^ According to Faith's phylogenetic alpha diversity study, the blood microbiome of rosacea patients differed somewhat from that of controls. The relative abundance of Fusobacteriaceae and Chromatiaceae families was substantially greater in the blood of patients with rosacea. Fourteen bacterial genera showed different abundance among patients with rosacea and controls. The Rheinheimera genus differed increasingly between groups, with rosacea patients having a much higher abundance.^40^ Recently, Shah et al. also discovered two variations (Phylum and Class level) in the blood bacterial DNA composition of patients with chronic kidney disease (CKD) vs. healthy at the phylum level (Proteobacteria: 61% in CKD vs. 54% in healthy), and the class level (Gammaproteobacteria: 45% in CKD vs. 38% in healthy). Even though these changes were statistically significant, their size was minor. However, investigations have shown that profiling 16S rDNA in the blood of CKD patients is a more difficult task, after accounting for a large number of comparisons and potentially confusing clinical factors.^41^ Another case‐control study was conducted regarding 15 age‐ and sex‐matched healthy vs. 28 women patients. They determined the microbiome in the blood using bacterial 16S ribosomal DNA sequencing. There are 216 genera, 12 phyla, 26 classes, 115 families, and 54 orders, among the 1352 operational taxonomic units (OTUs) were found. Proteobacteria, Actinobacteria, Bacteroidetes, Candidatus Saccharibacteria, and Firmicutes have mean relative loads of 77.56 percent (predominated), 13.23%, 3.98 percent, 3.05 percent, and 1.28 percent, respectively, among the 10 known phyla.^42^ This result followed what has previously been discovered in healthy people,^25^ as blood microbial community composition in CVD subjects is shown (Table 1). In this study, alpha diversity was not significant among both groups, although, between Rheumatoid arthritis patients and the control group, a significant variation was found in beta diversity.^29^ Obesity, diabetes, heart failure, neurodegenerative diseases, liver disease, cancer, and hematologic disorders all have bacterial components that may be detected in the blood.^25^ Inactive forms of many bacteria, including known pathogens, can be found in the blood and within RBCs. In this regard, thanks to recent developments in bacterial culturing methods, microscopy, and NGS approaches, we can better evaluate microbiota in both health and disease.

**TABLE 1:** Blood microbial community composition in cardiovascular disease (CVD) subjects

Samples	Materials	Method	Major finding	Reference
48 VHD, 35 CHD, 50 IHD, and 45 controls	Whole blood	16S rDNA analysis	All CVD patients had predominant *Staphylococcus sp*., while VHD patients had more bacteria detected than CHD and IHD patients	21
9‐year follow‐up of 3936 individuals without obesity or diabetes	Peripheral leukocytes	16S rDNA	Proteobacteria has a positive correlation with cardiovascular events, while Eubacteria has an inverse correlation	16
31 CVD patients and 10 controls	Whole blood	16S rDNA amplicon sequencing (Ion Torrent PGM)	In CVD patients, there is a higher in *Pseudomonadaceae* and a lower in *Staphylococcaceae*, Gamma Proteobacteria, and Bacillales	46
80 CVD patients and 40 controls	Blood plasma	16S rDNA amplicon sequencing	The globin/16S rRNA gene proportion was observed elevated in patients with CVD compared with controls. Actinobacteria predominated in CVD patients, whereas Proteobacteria predominated in healthy	17
727 incident stroke patients and 1312 incident coronary heart disease patients	Whole blood	From 1987 to 2017, Atherosclerosis Risk Communities research were analyzed	Infections in both inpatient and outpatient were linked to an increased risk of cardiovascular disease	51
49 healthy, 100 patients STEMI patients, and 50 stable CAD patients	Peripheral blood leukocytes	Next‐generation sequencing of the 16S rRNA amplicon (Illumina HiSeq)	In STEMI patients, there is an increase in gut microbial translocation, especially of Streptococcus spp., Bacteroides, and Lactobacillus, as well as (LPS and D‐lactate) bacterial metabolites	53
103 non‐MI individuals and 99 MI patients	Whole blood	16S rRNA amplicon (Illumina MiSeq)	In MI patients, lower cholesterol‐degrading bacteria and increased 16S rDNA concentration were linked to blood cholesterol levels, and the Caulobacterales order and Caulobacteraceae family were both considerably reduced	54

Abbreviations: CAD, coronary artery disease; CVD, cardiovascular disease; MI, myocardial infarction; STEMI, ST‐segment elevation myocardial infarction; VHD, valvular heart disease.

## 5 BLOOD MICROBIOTA AND CARDIOVASCULAR DISEASE

Previously numerous researchers have examined the impact of the microbiome in the progression of various disorders, with the majority of them focusing on the intestine microbiome.^43^ However, recently several studies led to explore the link between the circulating microbiome and disease in humans.^24^ A study reported that bacteria from the oral or the gut had been detected in atherosclerotic plaques, and atherosclerotic plaque contains substantially more Proteobacteria and fewer Firmicutes.^44^ Indicating that the filtering system is selective, allowing only some bacteria into the bloodstream.^14^ By contrast, the phylum Proteobacteria has been found in a lower proportion in stool samples, while the Bacteroidetes and Firmicutes taxa were the most diverse.^45^ In addition, a 6–9 year follow‐up analysis revealed for the first time that the circulating microbial community structure could predict the development of diabetes.^14^ After one year, the researchers found lower bacterial DNA and higher Proteobacteria phylum in the blood of people who had cardiovascular (CV) complications during follow‐up duration. Amar et al. revealed the Eubacteria and Proteobacteria tertiles hazard ratio for a primary outcome. Volunteers who had the decreased proportion of Eubacteria and the increased proportion of Proteobacteria had a 3.7‐fold higher risk of CV events than those who had the elevated levels of Eubacteria and the reduced level of Proteobacteria (*p* = 0.003).^16^ In 2013, Rajendhran et al. used 16S rRNA sequencing to assess microbial profiles in whole blood samples from CVD people and normal individuals (barcoded ion sequencing); Proteobacteria were on the overhead, whereas Firmicutes were on the decreased, despite at phylum level, no variation was observed. The same researchers discovered a notable increase in Pseudomonadaceae and a decrease in Staphylococcaceae, Gamma Proteobacteria, and Bacillales at a lower taxonomic level using shotgun metagenome sequencing.^46^ Dinakaran et al. demonstrated increased bacterial DNA concentration and microbiome variation in the CVD patients' blood. The study revealed that Actinobacteria phylum was highly enriched in healthy subjects while Proteobacteria were predominant in patients. The researchers also discovered that in the circulating virome of CVD patients, bacteriophages (Pseudomonas, Rhizobium phages, and Propionibacterium) were abundant, whereas eukaryotic viruses predominate in healthy people.

In contrast, a longitudinal study discovered a nonsignificant increase in Proteobacteria proportion and the converse relationship between Eubacteria 16S rRNA and CVD events such as myocardial infarction (MI).^17^ Although, several studies demonstrated that CVD patients had a high proportion of microbial DNA and Actinobacteria/Proteobacteria in their blood plasma.^12^ Moreover, the high abundance of Proteobacteria has also been found in atherosclerotic plaques, blood, gut, and many chronic inflammatory disorders, such as chronic lung diseases, metabolic syndrome, inflammatory bowel disease, and CVDs.^47^, ^48^ This suggests that inflammation caused by gut bacterial translocation into the blood is a common underlying mechanism in various disorders, resulting in pleomorphic microbial alterations in the blood.^49^ The rise in Proteobacteria in chronic inflammatory disorders is likely due to being released into the bloodstream after cell death in the indicated organs. Moreover, it has already been established that lipopolysaccharides are the major component of Proteobacteria, which play a key role in the onset of CVD.^41^ With this background, lipopolysaccharides are associated with a significantly increased risk of CVD produced by Proteobacteria, suggesting that in the onset of metabolic disorder, Proteobacteria might play a crucial role.

Additionally, the role of microbes in CVDs has been focused on pathogen‐related complications, such as pericarditis, endocarditis, rheumatic carditis, and myocarditis.^50^ Porphyromonas gingivalis, Enterococcus species, S. aureus, Chlamydia pneumoniae, Streptococcus species, Trypanosoma cruzi, Klebsiella, Helicobacter pylori, Escherichia coli, herpes simplex virus, and cytomegalovirus are the primary pathogens implicated in infections connected to CVD.^12^ Bacteremia and sepsis are caused by the proliferation of bacteria in the blood, resulting in death. Furthermore, the latest study of 1312 newly diagnosed CAD and 727 newly diagnosed patients with stroke discovered that pathogen infection is a significant predictor of CVD risk, mainly stroke.^51^ Microbes in the bloodstream are the leading cause of the microbial invasion of atherosclerotic plaques, which contributes to inflammation and CVD.^52^ In addition, investigations have discovered that bacterial species, mainly Staphylococcus species, are detected at higher levels in blood samples from patients with IHD, CHD, and VHD.^12^

Furthermore, Zhou et al. revealed that the breakdown of the intestine obstacle made up of tight junction proteins caused microbial translocation from the stomach to the blood in ST‐segment elevation myocardial infarction (STEMI) patients and mouse models. There was a rise in the discovery of intestinal bacteria in STEMI patients (Lactobacillus, Bacteroides, and Streptococcus).^53^ Amar et al. revealed that many (Norcardiaceae, Aerococcaceae, Gordonia, Propionibacterium, Chryseobacterium, and Rhodococcus) are present in the blood of our control and patients, and curiously, the relative proportions of all of them are decreased in MI patients. Furthermore, both statistical techniques revealed that the Caulobacterales order and the Caulobacteraceae family were significantly lower in the myocardial infarction patients, and their presence in the blood of MI patients appeared to be negatively associated with left ventricular ejection fraction (LVEF) at inclusion.^54^ Studies on blood microbiota in other diseases are described (Table 2). The data could not sufficiently explain the microbial community signature characteristics. However, various genera and species reported in the results were not always the same. Hence, the microbial community structure of blood is more intricate, necessitating a more detailed investigation.

**TABLE 2:** Studies on blood microbiota in other diseases

Study population	Material	Method	Major finding	Reference
119 diabetic and 480 nondiabetic	Whole blood	Cultured in aerobic and anaerobic broths separately	Diabetic patients have higher Klebsiella and Staphylococci	14
9‐year follow‐up of 3280 individuals without obesity or diabetes	Peripheral blood leukocytes	16S rDNA sequencing	In a 9‐year follow‐up, people with elevated 16S rDNA concentration in their blood developed diabetes regardless of other risk factors. Ralstonia spp. has a high prevalence in people who have diabetes	14
50 diabetics and 50 nondiabetic individuals	Blood plasma	16S rDNA sequencing	Diabetes patients had high 16S bacterial rRNA content, with Clostridiumcoccoides and the Atopobidum cluster being especially abundant	35
Community‐acquired (CA) Staphylococcus aureus bacteremia patients	Whole blood	From 2000 to 2011, a population‐based medical database was analyzed	CA‐SAB infection is more likely in people who have diabetes	35
58 Parkinson's disease (PD) patients and 57 healthy controls	Whole blood	16S rRNA sequencing	Genera Cloacibacterium and Isoptericola were found to be highly expressed in PD patients, while genera Paludibacterium and Saccharofermentans were found to be positively correlated with disease duration	35
20 healthy controls and 20 nondiabetic patients with chronic kidney disease (CKD)	Buffy coat samples	16S targeted metagenomic sequencing	The CKD group had higher levels of the Proteobacteria phylum, Gamma Proteobacteria class, and Enterobacteriaceae and *Pseudomonadaceae* families than the controls	41
50 diabetes mellitus and 100 nondiabetes Mellitus patients	Blood plasma	Next‐generation sequencing of the 16S rRNA amplicon	Bacteroides spp. were found to have a negative link with diabetes, but Sediminibacterium spp. had a positive relationship	40
10 subjects with rosacea patients and 30 healthy adults	Whole blood	16S rRNA gene sequencing	At the family level, *Fusobacteriaceae* and *Chromatiaceae* were the most common, and at the genus level, Rheinheimera was the most abundant in rosacea patients' blood	40
A total of 28 female patients and 15 age‐ and sex‐matched controls were included in the study	Blood	16S rDNA sequencing	The prevalence of the Proteobacteria phylum, genera Pelagibacterium, Halomonas, Aureimonas, Chelativorans, and others were increased in rheumatoid arthritis patients as compared to healthy	42

## 6 ORIGIN OF BLOOD MICROBES

The microbiota in the blood is still debatable whether it is indigenous or endophytic. Bacteria in the blood result in bacteria translocating from other parts of the body, primarily the gastrointestinal system (GIT).^25^ Indeed, bacterial translocation from the GIT, particularly through the intestinal epithelial mucosa, has been associated with the pathophysiology of diabetes, CVD, hematological disorders, and cirrhosis.^55^, ^56^ Other ways could enhance the passage of gut microbes into the blood even if the intestinal epithelial barrier is not disrupted. Dendritic cells (mammalian immune system antigen or accessory cells) can absorb microorganisms and transport them across the intestinal epithelium^57^ or with the help of intestinal mucus‐secreting goblet cells.^58^ Intestinal M cells (mucosa‐associated lymphoid tissues specialized epithelial cells) may also play a role in bacterial translocation from the intestinal lumen to the blood circulation system.^11^, ^59^

**FIGURE 2:**
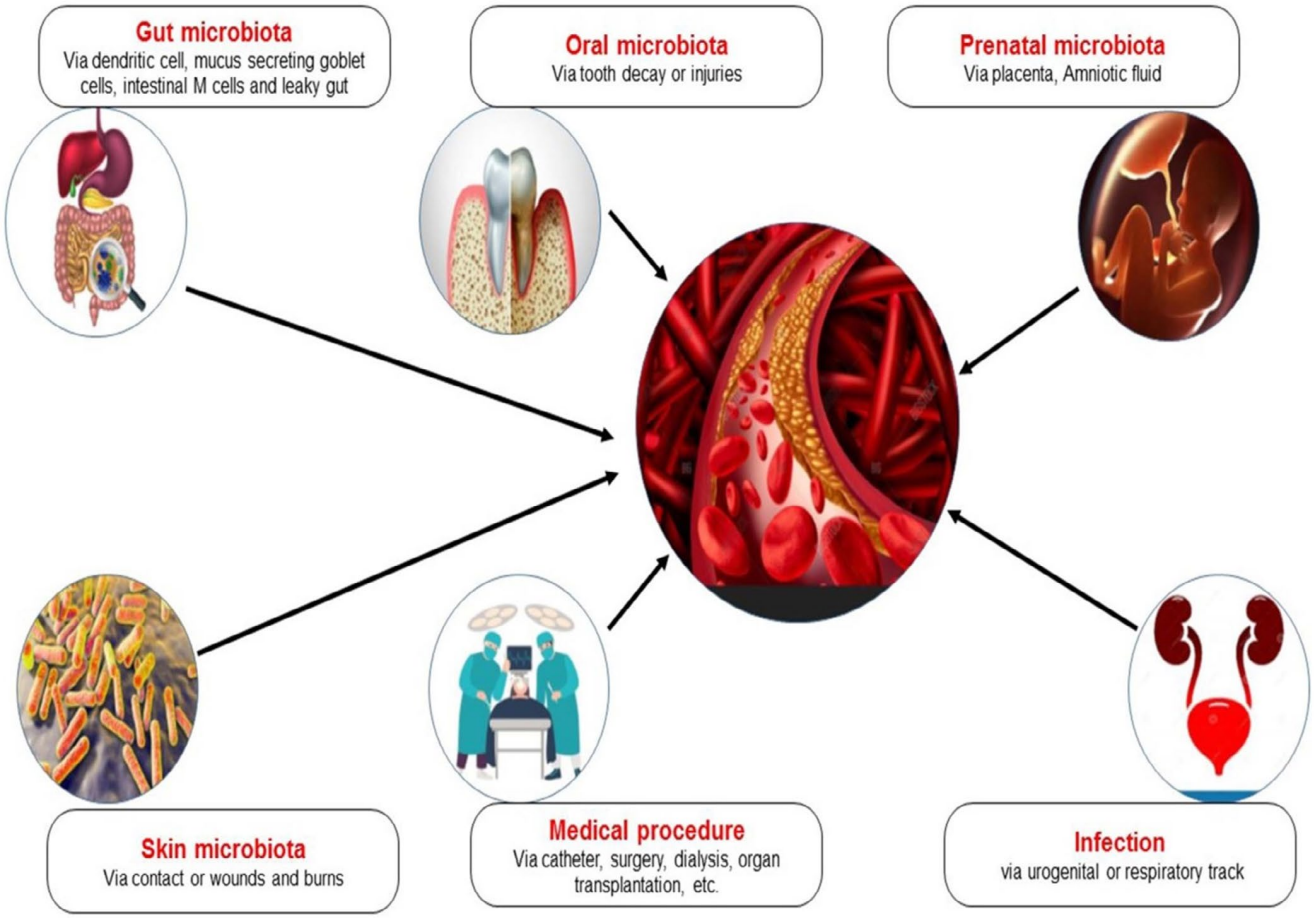
The human blood microbiota. Origins and portals of entry of microbes into the blood

In addition, a study compared healthy people's microbial DNA to microbiome data from the Human Microbiome Project. The study revealed that the blood microbiome is similar to oral and skin microbiota, whereas it was significantly different from the gut microbiota. Although bacterial transmission into the blood is unusual, it might take place more often in healthy humans, supporting recent findings of a healthy human blood microbiome (HBM).^60^ Furthermore, another important origin of microbial translocation into the blood circulation from a microbiome‐rich source leads to barrier breakdown, infection, and trauma.^61^ Bacterial translocation into the circulation is facilitated by daily activities such as biting, tooth cleaning, and flossing.^62^, ^63^ Periodontitis, a common inflammatory condition that affects more than half of the population over the age of 50, results in the breakdown of tooth‐supporting structures, and the deepening and ulceration of periodontal pockets, allowing bacteria to enter the bloodstream.^64^, ^65^ The human blood microbiota, origins, and portals of entry of microbes into the blood are presented (Figure 2). However, the exact origin of the human blood microbiome is still not confirmed. Further detailed research is required to explore the origin of blood microbiota.

## 7 CIRCULATING MICROBES AS A BIOMARKER AND TARGET FOR CARDIOVASCULAR DISEASE

**FIGURE 3:**
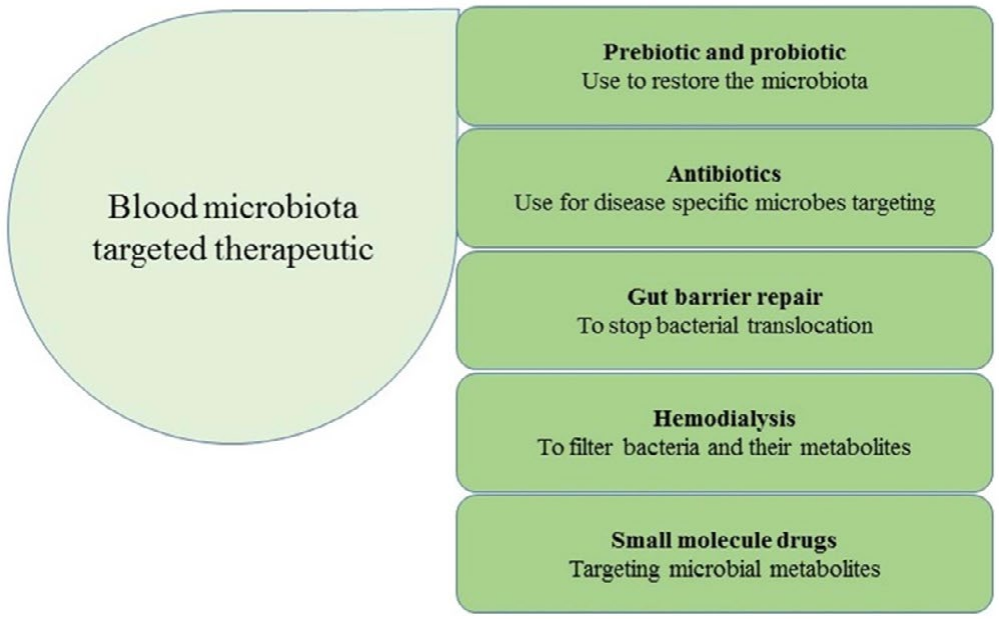
The blood microbiota and circulating metabolites are exploited as therapeutic targets for cardiovascular diseases

To investigate variation in the circulating microbiome and their metabolites, sophisticated techniques, such as NGS and metabolomics, are currently being used. In addition, biosensors and nanosensors for detecting specific bacteria or their metabolites will make incorporating these biomarkers into routine clinical examination much more accessible. As a therapeutic alternative, many treatments have been used to reinstate the circulating microbiome and metabolites, as shown in Figure 3. To restore GIT microbiota, which could have a significant impact on the circulating microbiome and metabolites. In this respect, prebiotics (dietary modifications),^66^ bacterial probiotics,^67^ and antibiotics^23^, ^68^ have been used to repair the gut barrier and restore the altered blood microbiota.^69^ Kidney dialysis, a frequent renal failure treatment, may be a therapeutic option for rebuilding the blood microbiota in diseased subjects. Gut bacteria and their metabolites can be selectively filtered using hemodialysis membranes. Such as, indoxyl sulfate has been successfully removed from patients with advanced kidney failure using the oral charcoal adsorbent (AST‐120).^69^ Drugs that target host mediators, microbiota, and their metabolites are currently being developed, with some being tried in clinical trials.^70^, ^71^ TMAO has been identified as the most promising therapeutic target for CVDs.^72^

## 8 LIMITATIONS AND FUTURE PERSPECTIVES

Blood is no longer thought to be a germ‐free zone. Numerous studies reported that blood circulation appears to be one of the most important bacterial habitats in the human body. The majority of clinical studies compare the blood microbial composition of the patient with healthy controls. Recent studies have offered a useful characterization of the blood microbial profile in patients with CVD; however, descriptive data remain a challenge. The biggest challenge we face is identifying a particular blood microbiota‐based target for preventing CVD. In this regard, most research uses just blood samples, making it impossible to determine whether a section of the blood microbiome differs in patients who have CVD before or after the disease. This information would be extremely useful in the development of CVD prediction biomarkers and/or therapeutic targets to avoid CVDs. In addition, despite its many advantages, such as high sensitivity and exhaustivity, the molecular approach based on the quantification and identification of bacterial DNA is unable to separate DNA from living bacteria from DNA originating from bacterial breakdown. Even if this is not a barrier for biomarker development, it makes understanding the mechanisms underlying blood microbiome diversity in CVD patients more difficult.

Furthermore, large sample size and participants from multiple locations should be included in future studies. Also, conducting a broad cohort study or a translational study to understand further how the blood microbiota directly contributes to CVD may take a little longer. Furthermore, studies speculated that bacteria in the blood came via leakage in the gastrointestinal tract. In contrast, others suggested that the microbiome may come from the oral tract or skin and disperse into the bloodstream when these protective barriers are breached.^11^ However, the above studies reported a high level of phylum Proteobacteria detected in the blood of CVD patients while the mechanism and pathogenic role of Proteobacteria in CVD remains to be explored. The existence of the microbiome in the blood was found to be more closely linked to CVD clinical features. As a result, the load and variation of the circulating microbiome are affected by the disease's clinical condition and immunological status. In this regard, obesity, age, smoking, gender, alcohol use, genetic predisposition, hypertension, and diabetes mellitus have an impact on the blood microbiome of CVD patients. Aside from these characteristics, decades of study have shown that GIT and oral cavity disorders have a substantial influence on the circulating microbiome in CVDs. The use of the intravenous drug, implantable cardiac devices, and prosthetic devices addiction may introduce circulating microbiota from outside sources, leading to CVD problems. As a result, explaining these changes in the structure of blood microbiomes will help us better understand the role of the microbiome in the pathology of CVDs, and the possible involvement of microbiome from various tissues will be explored further.

## 9 CONCLUSION

Blood is no longer considered a sterile environment, and multiple lines of evidence suggest that it is one of the major microbial niches of the human body. It is significant to remember that the presence of live bacteria in the blood cannot be directly linked to the detection of microbial DNA/RNA in the circulation. The blood microbiome and metabolome undergo significant alterations during CVD, suggesting that they may play a role in the disease's etiology and progression. Subsequently, to establish the blood microbiota and its metabolites as biomarkers and therapeutic targets in CVD, further study is needed to understand better the molecular mechanisms of microbial translocation and their physiology in disease development.

## CONFLICT OF INTEREST

The authors declare that they have no competing interests.

## AUTHOR CONTRIBUTIONS

Concept: Ikram Khan. Review: Imran khan. Figures preparation: Zhou Jianye, Zhang Xiaohua, Murad Khan, Mian Gul Hilal, and Mian Adnan Kakakhel. Funding acquisition: An Lizhe and Li Zhiqiang.

## CONSENT FOR PUBLICATION

All the authors agree to publish this paper.

## Data Availability

All processed data used in this study are included in the article.
